# Short-term forecasting of Indonesia electricity generation using MATLAB based on NARX neural network

**DOI:** 10.1371/journal.pone.0340268

**Published:** 2026-02-04

**Authors:** Nicholas Pranata, Fahmy Rinanda Saputri

**Affiliations:** Department of Engineering Physics, Universitas Multimedia Nusantara, Jl. Scientia Boulevard Gading, Curug Sangereng, Serpong, Kabupaten Tangerang, Banten, Indonesia; Southwest University of Science and Technology, CHINA

## Abstract

Electricity consumption, production, and supply based on fossil fuels have increased due to population growth, urbanization, and technological development, leading to environmental damage in countries like Indonesia. In response to this issue, electricity forecasting is essential. This study applies to a Nonlinear Autoregressive with Exogenous Input (NARX) neural network to forecast one year ahead of electricity generation using MATLAB. Two algorithms are used for comparison: Levenberg-Marquardt and Bayesian Regularization. The data is classified using a standard method of 70%−30% split, with 30 hidden layers and a standard delay of 2-time steps. The results show that both algorithms achieve an R² value above 0.9 and a Mean Absolute Percent Error (MAPE) of under 3%, with the Levenberg-Marquardt algorithm demonstrating marginally superior performance. These results indicate that the model provides valuable insights for forecasting annual electricity generation in Indonesia over a short timeframe.

## 1 Introduction

Electricity has become the primary source of global development, significantly affecting human activities. It supplies power to appliances and communication technologies across various sectors while also influencing the economic aspects on both individual and national scales [1]. As consumption, production, and supply increase due to population growth, urbanization, and technological development, the use of fossil fuels as primary energy sources rises significantly [2,3]. Climate change has heightened the importance of energy management and the implementation of renewable energy sources to address this challenge. Consequently, electricity forecasting has become essential for reducing unnecessary electricity consumption. By effectively managing the generation and distribution of electricity, utilities can better plan and operate in accordance with actual requirements [1,4,5]. This process can reduce greenhouse gas emissions damage while also helping to make important decisions regarding load switching, voltage control, network reconfiguration, and power markets [3,6].

Estimating a higher load than the actual demand leads to excessive activation of the power supply, resulting in unnecessary energy intake and reserves. Conversely, underestimating the load may result in an insufficient supply [2]. Effective management of both load and demand, supported by accurate forecasting, is crucial for the electricity market to meet its needs reliably. This approach aids in economic management, reduces operational costs, enhances electricity security analysis, and improves scheduling and maintenance planning. Ultimately, it ensures that the supply is stable and aligned with demand [1,4,5].

Accurate forecasting can stimulate economic growth by utilizing data and statistics to predict trends from the nonlinear data of power generation [4]. Load forecasting traditionally utilizes correlations between historical values and the forecasted value. This encompasses methods such as time series analysis, load derivation, regression analysis, and exponential smoothing [5]. However, these simple structural methods do not account for various load factors, leading to low prediction accuracy. Therefore, the integration of artificial intelligence (AI), specifically machine learning has become intriguing, resulting in numerous studies on electricity forecasting [5].

Several studies on electricity forecasting utilize machine learning methods such as Artificial Neural Networks (ANN), Fuzzy Time Series (FTS), Adaptive Neuro-Fuzzy Inference Systems (ANFIS), Least Squares Support Vector Machines (LSSVMs), Long Short-Term Memory (LSTM), and Autoregressive Integrated Moving Average (ARIMA) [7–10]. In association with electricity consumption in a specific region, the forecasting approach based on AI must be developed as accurately as possible to predict electricity demand and load which can reduce national operational price and in the long-term, boosting economic growth. While machine learning (ML) models offer significant benefits, particularly in improving energy efficiency through forecasting, there are several challenges and limitations that need to be addressed. One major issue is that ML models often struggle to manage complex and dynamic building environments. This challenge emphasizes the necessity for enhanced data sources to improve the accuracy of these models [11].

In the current context, this study specifically focuses on the case of Indonesia. Indonesia serves as the largest archipelago country globally [12]. As a developing country, Indonesia’s energy demand is growing rapidly, while the use of its primary generated sources, specifically fossil fuels, is in critical condition [13]. From 2015 to 2024, the per capita electricity consumption increased from 918 KWh to 1,408 KWh, clearly indicating a rising trend [14].

The government has introduced several programs aimed at reducing energy consumption from fossil fuels. However, various challenges hinder progress in this area. Therefore, it is essential to employ forecasting methods, particularly through the integration of AI, to support energy management between generation and distribution. As a developing country that heavily relies on fossil fuels, Indonesia faces potential issues related to resource depletion in the future. Over the past five years, electricity forecasting studies specific to Indonesia have been conducted, yielding significant results for both the country as a whole and specific regions. These forecasts utilize a variety of machine learning models, with a strong emphasis on neural network models based on several software, such as MATLAB, Python, etc.

MATLAB, abbreviated by Matrix Laboratory, is a matrix-based software made for programming, analysis and computation of mathematics and engineering [15]. This software, made by 1970 by Cleve Moler, is widely recognized for its ability to perform analytical operation, data visualization, modelling, and simulation in which are still developing and adapting periodically [15,16]. MATLAB supports research in many fields such as engineering, biology, finance, etc. [16]. This software offers ease of use, versatility, and powerful analytical capabilities, along with prebuilt tools and add-ons. It supports reproducible research, algorithm development, and time savings while providing a multi-paradigm numerical computing environment and data visualization tools. In engineering, it is applied in signal and image processing, control systems, computational fluid dynamics, robotics, electrical circuit design, materials science, biomedical applications, communication systems, and artificial intelligence. The software enhances machine learning and deep learning efforts through additional toolboxes. Several studies have employed it for energy consumption forecasting, analyzing data trends [17].

A study uses fuzzy logic and artificial neural network to forecast electricity demand in the Jakarta distribution grid system from 2016–2019 data with an error range of 2–3% [18]. Another study utilizes fuzzy logic to forecast energy consumption from 2020 to 2025 based on Rayon West Semarang historical data from 2015 to 2019 with an error value of 0.17% [19]. With similar models, a study forecasting a long-term annual electricity consumption for East Kalimantan Province using 2010–2018 data for 2019–2028 prediction with a Mean Absolute Percentage Erro (MAPE) value of 3.8% [20]. Finally a study forecast Riau’s electricity consumption from 2024 to 2027 using data from the previous 10 years with a MAPE value of 4.315% [21].

The existing studies, including the previous research, focus on fuzzy logic and neural network to forecast electricity in a certain location. In this study, Indonesia’s electricity generation data from 1988 to 2023 will be used to forecast one year ahead data which is 2024 using Nonlinear Auto-Regressive model with eXogenous input (NARX) Neural Network model, serving as the research purpose and gap. This paper also contributes to the achievement of several Sustainable Development Goals (SDGs) through energy management. The relevant goals include Affordable and Clean Energy (SDG 7), Sustainable Cities and Communities (SDG 11), and Climate Action (SDG 13).

## 2 Methods

### 2.1 NARX-NN model

**Fig 1:**
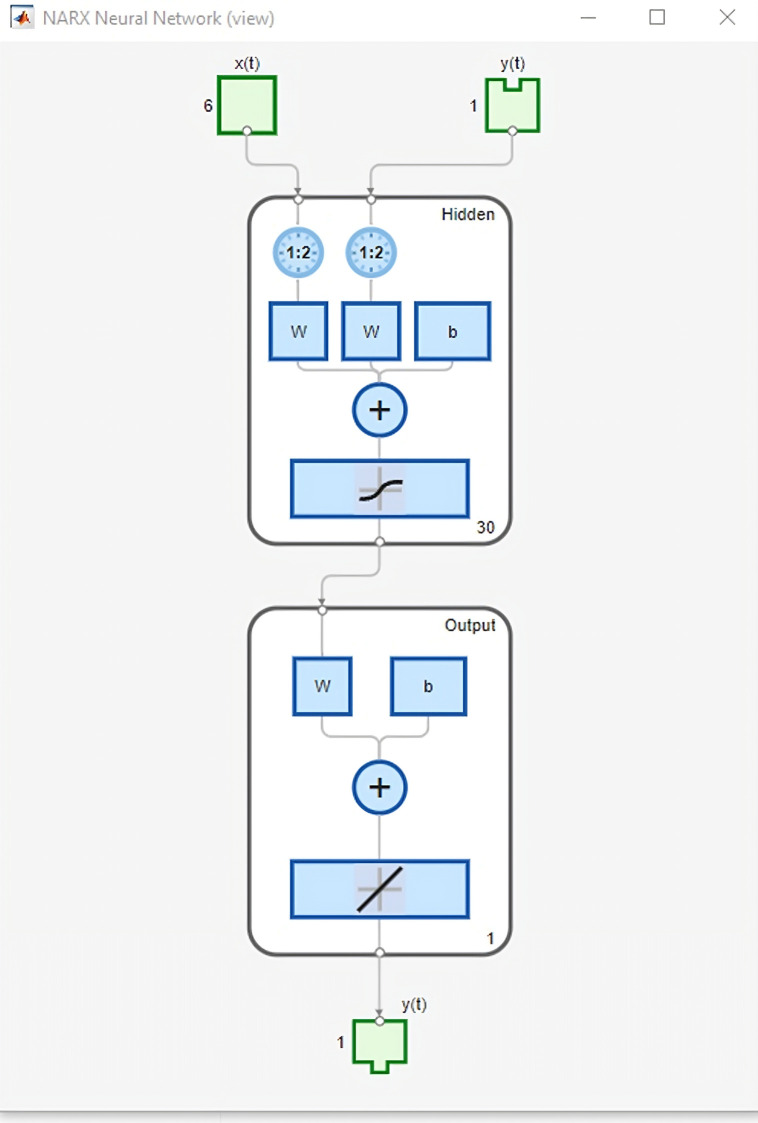
NARX structures.

This is how to start a subsection. Artificial Neural Networks are based on the human neural system, forming a network structure as a multilayer perceptron, which consists of an input layer, hidden layer, and output layer, as shown in Fig 1 [2,8]. Similar to neurotransmitters in a biological brain, each part of an artificial neural network sends signals among artificial neurons [22]. When a neuron gets a signal, it processes that information and may transmit it to other neurons. The links between neurons, referred to as “edges,” possess weights that modify through learning, influencing the intensity of the signal. A signal can only pass through an edge if it completely goes through. Artificial neurons are structured into layers, where each layer modifies the input data in different manners. Following the passage through several layers, the data transitions from the main layer to the last layer.

This study utilizes the NARX model which is capable in obtaining nonlinear relationships based on equation (1) [23,24].


$$y(t+\text{1})=f\left(y(t),\;y(t-\text{1}),\ldots ,\;y\left(t-n_{y}+\text{1}\right);u(t),\;u(t-\text{1}),\ldots ,u\left(t-n_{u}+\text{1}\right);w\right)=f(y(t);u(t);w)$$ (1)

In this context, u(t) and y(t) represent the input and output of the network, respectively, where (t) indicates the time step [22,24]. The parameters n_u_ and n_y_ refer to the input and output layers of the network, respectively, while the hidden layer is involved in the nonlinear function [22,24]. The f and w are the nonlinear function and weight utilized to predict the Multi-Layer Perceptron (MLP) [24]. This function utilizes a dynamic recurrent neural network that processes input data and feeds it back from the output to the function until convergence is achieved [22]. The NARX models are classified into two types, which are parallel architecture and series-parallel architecture. Parallel architecture can be shown in the equation (2) [24]. This architecture relies on the output or the forecasting results as the feedback for the input system, which is often used for long-term forecasting, shown in equation (2).


$$\hat{y}(t+\text{1})=\hat{f}\left(\hat{y}(t),\;\hat{y}(t-\text{1}),\ldots ,\hat{y}\left(t-n_{y}+\text{1}\right);u(t),\;(t-\text{1}),\ldots ,u\left(t-n_{u}+\text{1}\right);w\right)=\hat{f}(y_{p}(t);u(t);w)$$ (2)

Whereas the series-parallel architecture does not use forecasting results as the input, purely feedforward in which can be shown in equation (3).


$$\hat{y}(t+\text{1})=\hat{f}\left(y(t),\;\hat{y}(t-\text{1}),\ldots ,y\left(t-n_{y}+\text{1}\right);u(t),\;(t-\text{1}),\ldots ,u\left(t-n_{u}+\text{1}\right);w\right)=\hat{f}(y_{sp}(t);u(t);w)$$ (3)

The NARX model is widely used in various applications of time-delay neural networks, effectively addresses long-term dependency problems, and generalizes better than other artificial neural networks [22,24].

### 2.2 Data normalization

The data for this study is based on electricity generation from 1988 to 2023. The data selected input is based on the annual electricity generation from 1988 to 2017 for training and testing while the target data is used in the next 6 years from 1994 to 2023. The conventional method is used, which is 70% for training and 30% (15% for validation and 15% for test). The training procedures were prepared, and the input along with feedback data sequences were refined and structured. A normalization function was used on the input values to convert the data into a standard range, improving the fitting process for training. In the testing phase, the input and feedback values were transformed to a scale ranging from 0 to 1 before being returned. Aside from this, the hidden layers used in this cased in based on Heaton research that states for the number of hidden neurons to be 2/3 the size of the input layer added by the size of the output layer [25]. Since the input layers are the matrix 6x6 (taking 6 years from 1988) and the output layer is 6 for the last 6 years counting from 2023, which is based on the data structure approach by the study [26], the hidden layer is 30. Whilst the delay is set to be as default which is amounted by 2 steps.

### 2.3 Training algorithms

The training algorithm used in this study will be based on Levenberg-Marquardt (LM) and Bayesian Regularization (BR) method. The LM algorithm The LM algorithm was designed to approach second-order training speed without the necessity to compute the Hessian matrix as shown in equation (4) [22].


$$\Delta w={\left[J^{T}J+\mu I\right]}^{-\text{1}}J^{T}(w)e(w)$$ (4)

In which w represents the weight, $J^{T}J$ as Hessian matrix, J as Jacobian matrix, μ as learning constant (discover the minima in each iteration based on the error discovery), I as identity matrix and e as the vector of errors. Whilst the BR algorithm uses a single optimal weight vector (w) to produce the set of observed target data y = (y1, y2, …, yn) for the input x = (x1, x2, …, xn). This training can predict the posterior probability distribution of the weights given by the observed data as shown in the equation (5) [22].


$$P\left(w|y,\;x\right)=\frac{p\left(y|w,\;x\right)\;p(w)}{p\left(y|x\right)=\;\int p\left(y|w,\;x\right)\;p(w)dw}$$ (5)

In this context, P(w) represents the prior weight distribution, while P(y|w, x) denotes the likelihood function, which conveys information about w based on the data. The likelihood function is commonly represented as L(w)

### 2.4 Evaluation of performance

R^2^ is a determination coefficient used to analyze the best linear fit between the observed and predicted values given in equation (6) [22,27].


$$R^{\text{2}}=\frac{\sum {\left({\hat{y}}_{i}-y_{i}\right)}^{\text{2}}}{\sum y_{i^{\text{2}}}\left(\frac{\sum {\hat{y}}_{i^{\text{2}}}}{n}\right)\;}$$ (6)

In which y_i_ represents the observed values, ${\hat{y}}_{i}$ represents the predicted values and n represents number of observations. Whereas the measurement accuracy of forecasting is done by using Mean Absolute Percent Error (MAPE) value which is often used for time series forecasting. A very well performance has a value below 10% while the range of 10–20% shows well performance. The equation can be shown in equation (7) [23,27,28].


$$MAPE=\frac{\text{1}}{N}\sum_{t=\text{1}}^{N}\left|\frac{A_{t}-F_{t}}{A_{t}}\right|$$ (7)

In which N is the number of data, At is actual values at data time t, and Ft is forecast values at data time t.

## 3 Results and analysis

**Fig 2:**
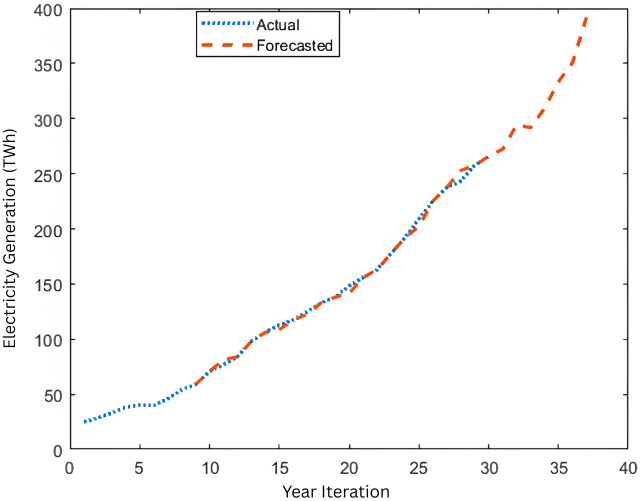
Indonesia Electricity Generation Forecasting (1988-2024) for Levenberg-Marquardt algorithm using NARX-NN model.

**Fig 3:**
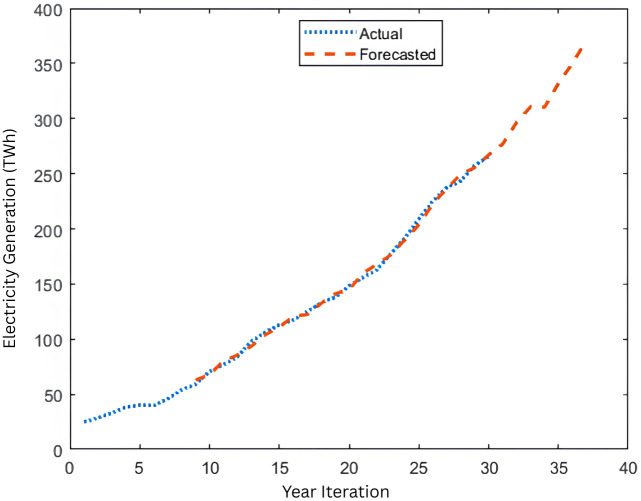
Indonesia Electricity Generation Forecasting (1988-2024) for Bayesian Regularization algorithm using NARX-NN model.

As mentioned priorly, this study uses NARX-NN algorithm in which is done using MATLAB 2024 (b) toolbox called Deep Learning Toolbox that provides several features, one such as Neural Time Series. In this study, the accuracy of the model is based on the standard error namely R^2^ and MAPE as discussed previously. The results can be shown in Figs 1–3 below.

**Fig 4:**
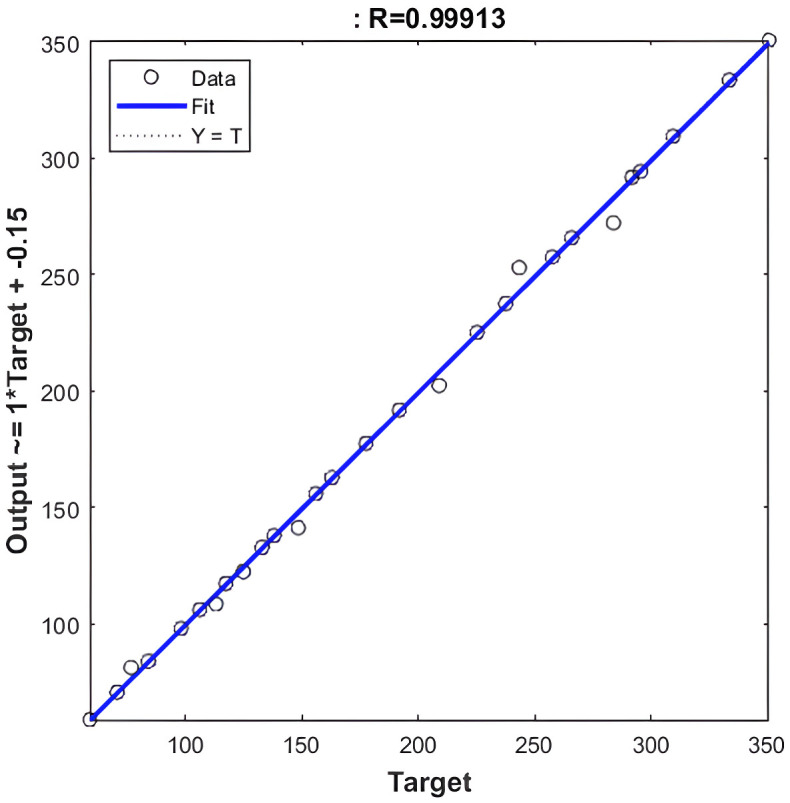
Regression value of NARX-NN for Levenberg-Marquardt algorithm.

**Fig 5:**
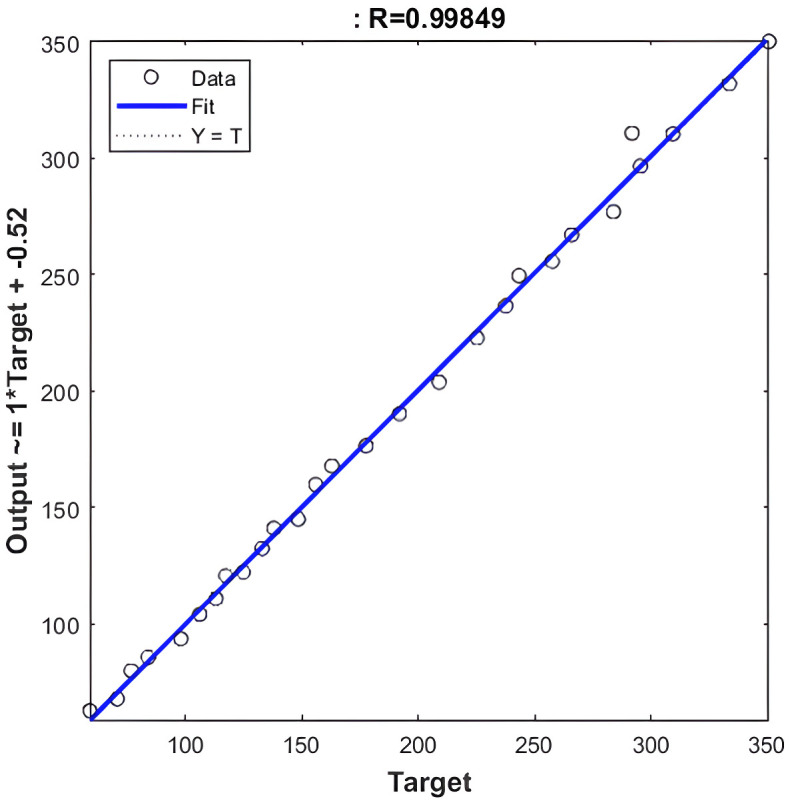
Regression value of NARX-NN for Bayesian Regularization Algorithm.

**Fig 6:**
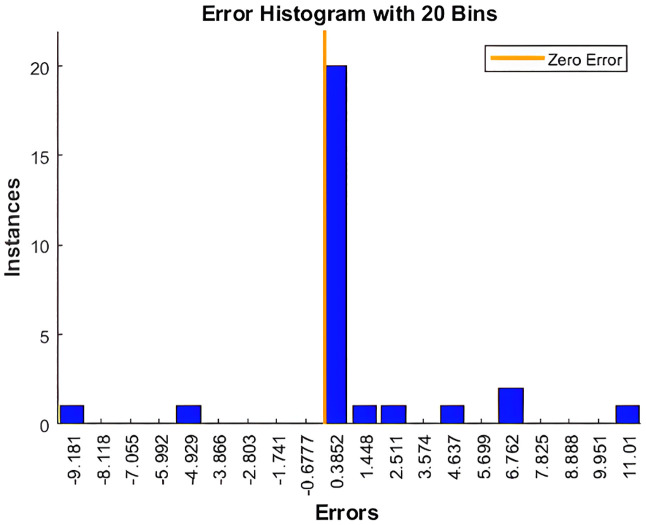
Error histogram of NARX-NN for Levenberg-marquardt algorithm.

**Fig 7:**
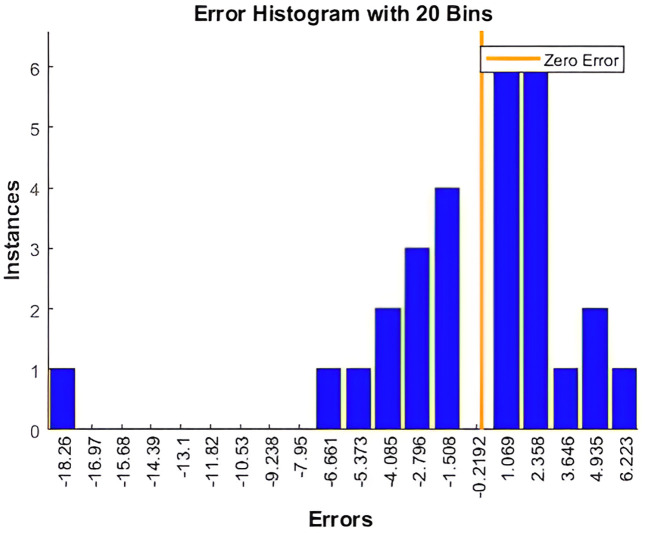
Error histogram of NARX-NN for Bayesian regularization algorithm.

Fig 1 shows the NARX structures while Figs 2 and 3 shows electricity generation graph for actual from 1988 to 2017 and forecasted data from 1996 to 2024 using Levenberg-Marquardt Algorithm and Bayesian Regularization Algorithm respectively. In this case the LM algorithm results show the data in 2023 (data number 35) by 350.069 TWH (TeraWatt Hour) whereas the BR algorithm shows the data in the same year by 350.106 TWH in which the actual data in 2023 is 350. 609 TWH. This shows that BR is closer to the actual data, showing more accuracy in forecasting. Whereas for the 2024 data as one step ahead forecasting results, the data for LM algorithm results amount by 391.641 TWH while the BR algorithm accounts for 371.127 TWH. Corresponding to the accuracy, based on R^2^ and error histogram, both algorithms’ results can be depicted in Figs 4–7.

Based on Figs 4, and 5 that shows the R-values based on Levenberg-Marquardt Algorithm and Bayesian Regularization Algorithm respectively, which are greater than 0.9, indicate that the training data were well fit, as illustrated in Figs 2 and 3 for both algorithms although LM shows slightly higher R-Values. Additionally, the error histograms presented in Figs 5 and 6 demonstrate that the analysis data from the BR algorithm performed better than that from the LM algorithm, as evidenced by more data points being closer to the zero error line. Finally, based on MAPE, the results for LM and BR algorithms, respectively, are 1.01% and 2.13%. From both R-Values and MAPE findings, LM is shown to be better since it has a higher R^2^ and smaller MAPE. However, the forecasted results show higher accuracy by comparing 2023 data for the actual and the predicted output. Therefore, it can be concluded that for both LM and BR algorithms are essential for comparison and analysis since both have a slight edge over each other. In terms of accuracy, BR is better while LM is faster. In general, the NARX-NN model can be recommended as an effective electricity generation forecasting tool which is seen from the results, although data are limited. Hence, several recommendations for future research include gathering more data for long-term forecasting, utilizing other machine learning models for comprehensive comparison, and considering the trial and error method to determine the best hidden layers to optimize the results.

## 4 Conclusions

This paper offers a research study on forecasting electricity generation in Indonesia, analyzing data from 1988 to 2023, by utilizing NARX-NN to project the generation for the one year ahead which is 2024. Employing two algorithms, Levenberg-Marquardt and Bayesian Regularization, along with a data classification through a standard method of 70%−30%, 30 hidden layers, and a standard delay of 2 steps, the training outcomes demonstrate precise electricity generation predictions in relation to the real data. This can be observed through the evaluation standards, which are R^2^ above 0.9 and under 3% for both algorithms, with the LM algorithm showing marginally superior performance, signifying an excellent result. The results from the LM algorithm indicate that the data for 2023 (data number 35) is 350.069 TWH (TeraWatt Hour), while the BR algorithm presents the same year’s data as 350.106 TWH, with the actual figure for 2023 being 350. 609 TWh. In contrast, for the 2024 data regarding one-step-ahead forecasting outcomes, the LM algorithm yields results totaling 391.641 TWH, whereas the BR algorithm reports 371.127 TWH.

Regarding model convergence, the LM algorithm needed the fewest iterations, and its reduction in time was quicker than that of the BR algorithm. Nonetheless, the overall performance of BR was anticipated to be stable and thorough. Overall, the results of the case study are promising and indicate that the NARX-LM and NARX-BR effectively predict annual electricity generation. Consequently, this kind of network heavily relies on the accessibility of training data, while the accuracy of predictions is influenced by the quality of the input data given to the training system. Thus, for future research, suggestions involve collecting additional data of electricity generation such as monthly data for long-term forecasting, employing various machine learning models for thorough comparison, and exploring the trial-and-error approach to identify the optimal hidden layers for enhancing outcomes.
